# Possible Isolated Pulmonic Valve Endocarditis in a Patient With Sickle Cell Disease: A Case Report

**DOI:** 10.7759/cureus.37043

**Published:** 2023-04-02

**Authors:** Kalaila Pais, Qasim Khurshid, Amir Shahbaz, Ahmed Brgdar

**Affiliations:** 1 Internal Medicine, Howard University College of Medicine, Washington, D.C., USA; 2 Internal Medicine, Howard University Hospital, Washington, D.C., USA; 3 Internal Medicine, Sheikh Zayed Hospital, Lahore, PAK

**Keywords:** echocardiogram, sickle cell anemia, vegetation, infective endocarditis, pulmonic valve

## Abstract

Pulmonic valve endocarditis is a rare and clinically elusive identity, commonly associated with congenital heart malformations and intravenous (IV) drug abuse. We describe a case of a 40-year-old male who has established sickle cell disease and presented with pain crisis, febrile episodes, and oxygen desaturation on room air. The clinical presentation and echocardiographic findings of a pulmonic mass were consistent with the diagnosis of pulmonic valve endocarditis. Due to the small size of the pulmonic valve vegetation, the patient was treated with antibiotics and discharged home on antibiotics and home oxygen.

## Introduction

Right-sided infective endocarditis (IE), commonly associated with intravenous (IV) drug use, intra-cardiac devices, and central venous catheters, accounts for only 5%-10% of all cases of IE. Furthermore, isolated pulmonic valve IE is rare and accounts for <2% of patients with IE, 14 with only 70 reports of isolated pulmonic valve IE published between 1979 and 2013 [1].

Homozygous sickle cell disease (SCD) is an autosomal recessive inherited hemoglobinopathy resulting from the presence of a mutated form of hemoglobin, hemoglobin S (HbS). The disease can be seen primarily in India, the Middle East, South Europe, and among Afro-Caribbean populations and has a prevalence of 5% [2]. The mutation of the beta globin chain of hemoglobin (Hb) results in the deformation of Hb in hypothermic, hypoxic, acidotic, or low-flow states, leading to an increased risk of patients developing a myriad of clinical presentations involving cardiopulmonary, vascular, and renal systems. This case report describes the hospital course of a homozygous HbS patient admitted due to a pain crisis with the subsequent finding of an isolated pulmonic valve mass on an echocardiogram.

## Case presentation

A 40-year-old male with a past medical history of SCD presented with a one-day history of generalized body pain rated as 10/10 in intensity. He denied vomiting, diarrhea, shortness of breath, cough, fever, and chills. Considering baseline hemoglobin of 8.5g/dL and a recent hospitalization for a pain crisis five months previously, a provisional diagnosis of vaso-occlusive crisis secondary to SCD was made. He did not have an established hematologist and had previously been managing his pain crises with ibuprofen. On admission, the patient’s blood pressure ranged between 146-168/74-99 mmHg until management with amlodipine besylate 5mg. The laboratory work is illustrated in Table 1.

**Table 1:** Blood Test Results TLC=total leucocyte count, Hb=hemoglobin, HCT=hematocrit, MCV=mean corpuscular volume, LDH=serum lactate dehydrogenase, Na=sodium. K=potassium, Cl=chloride,  HCO^3^=bicorbonate, BUN=blood urea nitrogen, Cr=creatinine, PCO2=partial pressure of carbon dioxide, PO2=partial pressure of oxygen, FiO2=fraction of inspired oxygen, SO2=oxygen saturation

Blood tests	Day 0	Day 3	Day 4	unit	Reference range
TLC	11.5	13.28	15.1	10^9^/L	3.2-10
Hb	8.2	8.1	8.2	g/dL	11.5-16
HCT	24.3	24.4	23.9		35- 50
MCV	75	75	75	fl	75-95
Reticulocyte %	12.5	12.8	11.8	%	
LDH	444	490	510	U/L	05-333
Haptoglobin.	<30	<30	<30	mg/dl	50-220
Na	138	131	133	mmol/l	136-145
K	4.3	4.8	4.6	mmol/l	3.5-5.0
Cl	105	103	101	mmol/l	98-107
HCO3	33	38	34	mmol/l	21-32
BUN	11	14	18	mg/dl	8-22
Cr	0.8	1.2	1.1	mg/dl	0.75-1.25
pH	7.32	7.34	7.39		7.35-7.45
PCO2	54	52	56	mEq/L	35-45
PO2	101	97	104	mmHg	75-100
FiO2	33	30	32		40-100
SO2	94	89	98	%	95-100
D-dimer			3.34	g/l	<0.50

**Figure 1:**
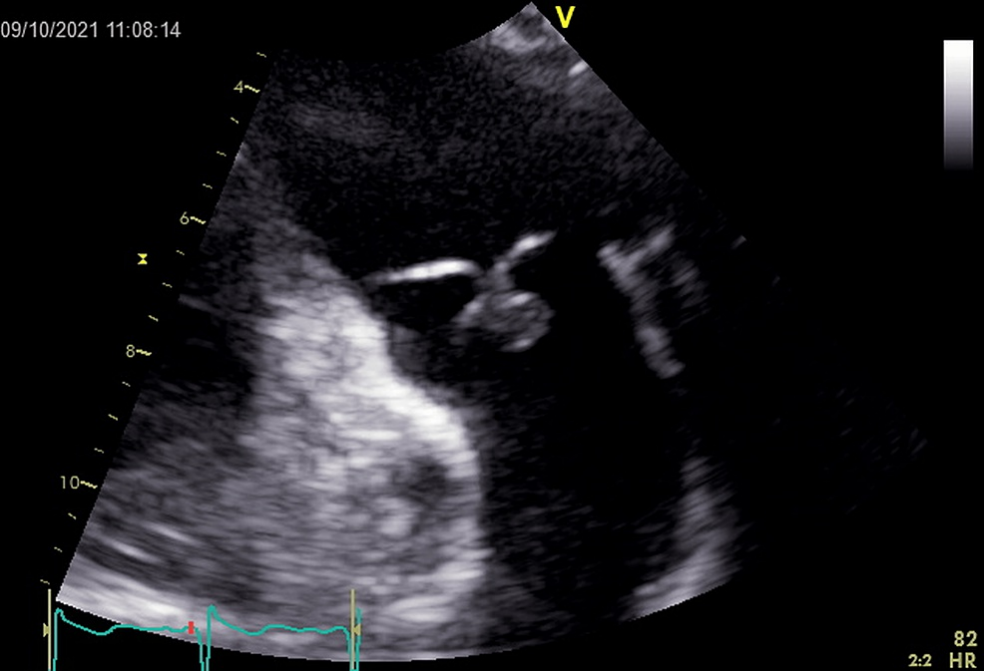
Transthoracic echocardiogram showing 0.7 X 0.9 cm mobile structure seen on the pulmonic valve leaflet

**Figure 2:**
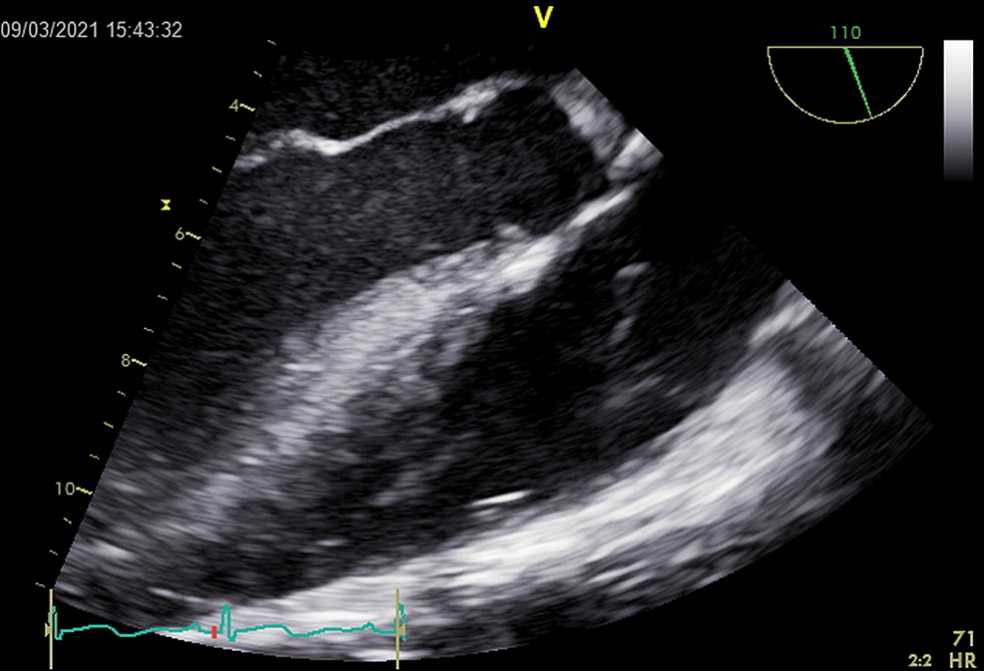
Transesophageal echocardiogram showing 1.1 X 0.5 cm vegetation noted on the pulmonic valve

Baseline tests showed normal serum electrolytes and renal function tests, complete blood count showed a total leukocyte count (TLC) of 10.5 × 10^9^/L. His initial chest X-ray was normal. On day three of admission, the patient had a febrile episode and a subsequent spike in TLC from 11.7 × 10^9^/L to 13.28 × 10^9^/L. Intravenous piperacillin was started, and blood cultures were drawn. On the fourth day of admission, the patient began to desaturate on room air and required 3L of oxygen by nasal cannula. Arterial blood gas (ABG) showed pH 7.39, bicarbonate (HCO^3^) 34 mEq/L, partial pressure of carbon dioxide (PCO2) 56 mEq/L, partial pressure of oxygen (PO2) 104 mmHg, fraction of inspired oxygen (FiO2) 32, oxygen saturation (SO2) 98%, chest X-ray showed bibasilar subsegmental atelectasis and chronic interstitial lung changes. To rule out a pulmonary embolism, a D-dimer and ventilation/perfusion (V/Q) scan were ordered. The D-dimer was high at 3.34, and the V/Q scan showed a low probability of acute pulmonary embolism. Additionally, urinalysis was negative for leukocytes and nitrites, and blood cultures x2 were negative for growth. A transthoracic echocardiogram (TTE) for assessment of pulmonary hypertension showed a 0.7 x 0.9 cm mobile structure on the pulmonic valve leaflet causing moderate pulmonary regurgitation. while the rest of the valves were normal (Figure 1). On day 10, a transesophageal echocardiogram (TEE) confirmed an isolated pulmonic valve mass, possible vegetation (Figure 2).

**Figure 3:**
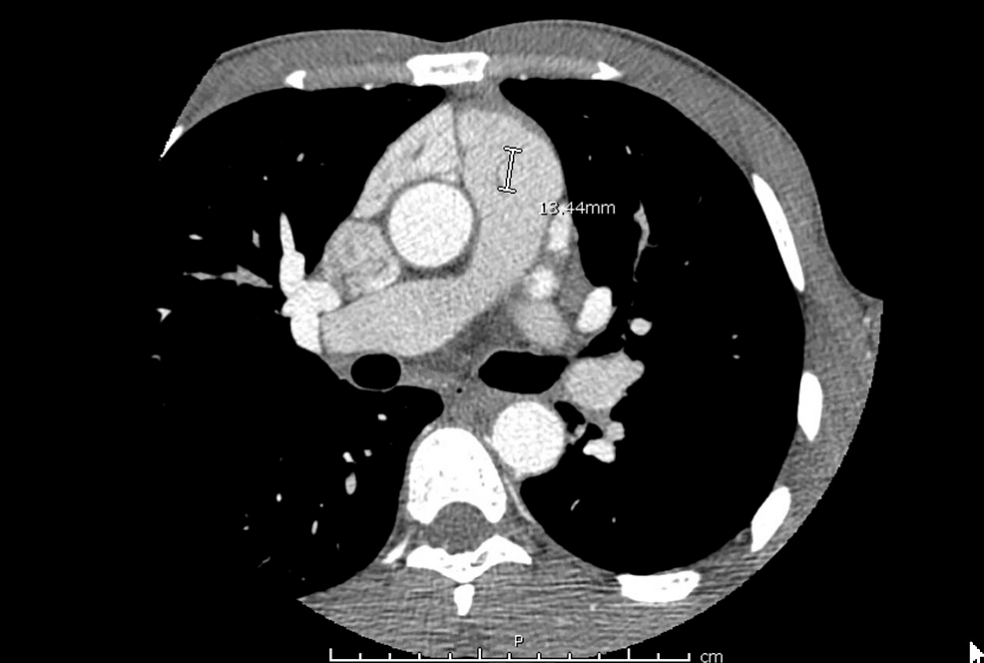
Cardiac computed tomography showing thin, curvilinear strand-like densities surrounding the pulmonic valve consistent with the findings of the prior echocardiogram suggesting vegetation likely resolving

Repeat chest X-ray showed no acute focal airspace disease, increased interstitial markings bilaterally, and an elevated left hemidiaphragm. Because of the possible vegetation, vancomycin (trough 15-20) and ceftriaxone 1g q 24 after consultation with infectious disease were started. Cardiothoracic surgery consultation established that the mass was too small to warrant surgical intervention. On day 15, the patient’s blood cultures x3 returned negative. A cardiac computed tomography (CT) on day 20 showed thin, curvilinear strand-like densities surrounding the pulmonary valve. This report was consistent with the findings of the prior echocardiogram suggesting vegetation, likely resolving (Figure 3). 

The patient failed the six-minute walk test: room air (RA) at 85%, Exertion on RA at 82%, and Exertion on 3L nasal cannula (NC) 95%. His discharged medications were amlodipine, folic acid, vitamin B1 100mg, Augmentin, Dilaudid, and MS Contin, and follow-up visits were scheduled to hematology, pulmonology, cardiology, and infectious disease outpatient clinics. 

## Discussion

Isolated pulmonic valve endocarditis is extremely rare, accounting for less than 1.5% of patients who suffer from IE [3]. The reasons for the scarcity of right-sided IE in comparison to those on the left side are differences in hemodynamic pressure gradients across valves, the differences in the incidence of congenital or acquired valvular abnormalities, and the fact that the right chambers of the heart have a lower blood oxygen content [4]. Complications owing to this rarity include initial misdiagnosis as a viral illness and subsequent delays in interventions [5]. Lack of signs related to the cardiac disease can contribute to delays in diagnosis, particularly in patients without traditional risk factors or concurrent involvement of other valves [6]. Moreira et al. state that the prognosis of isolated pulmonic valve endocarditis is determined by early diagnostic suspicion [3]. Patients tend to present with pulmonary symptoms such as cough, dyspnea, pleuritic chest pain, and hemoptysis [6].

As our patient had an established diagnosis of sickle cell disease, treatment of the pain crisis and exclusion of acute chest syndrome was the priority in his therapeutic management. Persistent complaints of shortness of breath, oxygen desaturation, febrile episodes, and an increase in white blood cell count resulted in a deeper look into his disease process.

The incidental finding of pulmonary valve mass on TTE conducted for assessment of pulmonary hypertension due to his unexplainable oxygen desaturation gives an initial clue about the possibility of pulmonic valve endocarditis. The modified Duke criteria define a “possible IE” as having one major and one minor criterion or three minor criteria. Our patient fulfills the criteria to have a “possible IE” in that he displayed one major (vegetation showed on echocardiography) and one minor criterion (fever > 38C).

Given the lack of display of other symptoms and negative blood and urine cultures, the importance of echocardiography in the diagnosis of this patient is evident. Li et al. 2000 state that TEE has improved the use of the Duke criteria in patients with “possible IE” and a negative TTE [7]. They advocate for TTE to be the initial test of choice in most patients but insist that TEE be the initial diagnostic test in patients with at least “possible IE” those with high suspicion of complicated IE, and those with prosthetic valves. This suggestion is agreed upon by Sakuma et al. 2020 who state that in patients with suspected infective endocarditis TTE and TEE should be used to provide complementary information [8]. Saremi et al. 2014 attest that cardiac CT and MRI are valuable diagnostic tools to assess the anatomy and function of the pulmonary valve and the diagnosis of conditions affecting the pulmonary valve [9].

Studies have shown that electrocardiogram-gated CT has a high diagnostic performance in detecting cardiac vegetation and perivalvular complications and is used in cases where TEE is nondiagnostic or contraindicated [8,10]. In fact, cardiac CT was incorporated into the Modified diagnostic criteria for IE in the 2015 European Society of Cardiology. In our case, the cardiac CT aided in the confirmation of “possible IE” as it revealed thin, curvilinear strand-like densities surrounding the pulmonary valve consistent with the findings of the prior echocardiogram, suggesting vegetations that were likely resolving. An alternative imaging tool is cardiac MRI, which can reveal the diagnosis of endocarditis through delayed contrast enhancement of the endothelial lining. However, while research on the use of cardiac MRI in the diagnosis of IE is ongoing, no studies have been conducted before 2015 [11].

For the management of patients with right-sided endocarditis, conservative treatment is usually the preferred route of choice as right-sided IE holds a more favorable prognosis than those found on the left side and is more likely to respond to medical therapy [12]. Surgical debridement or vegetation excision is indicated in certain instances like persistent bacteremia refractory to medical management, worsening destruction and incompetence of the valve, infection, and abscess formation [12]. In our patient, considering the small size of the mass, no indications for surgery [13], and high morbidity and mortality relating to his HbSC genotype, surgery was not performed [14].

One notable consideration is the recurrent lack of growth of organisms on multiple blood cultures and urine samples and nasal swabs seen in the workup of our patient. A study conducted by Miranda et al. in 2015 on patients aged ≥ 18 years seen from 2000 to 2014 who had a diagnosis of native and unequivocal involvement of pulmonic valve endocarditis found that the most common pathogens were Enterococcus faecalis and viridans group streptococci (in 22% of cases) [15]. Conversely, in a review of right-sided infective endocarditis, Staphylococcus aureus was found to be the most common causative organism [1]. In our case, we can consider the diagnosis of culture-negative bacterial endocarditis (CNBE). Skalweit et al. 2016 consider CNBE “one of the most challenging infectious diseases clinical syndromes both diagnostically and therapeutically” [16]. Definitive diagnosis needs a biopsy and treatment includes antibiotics and cardiac surgery.

Another point to consider is the lack of risk factors in our patient for IE. Predisposing factors for pulmonic valve IE include male gender, alcoholism, dental extraction, gonorrhea, liver or renal transplantation, bowel surgery, colonic angiodysplasia, central venous catheters, congenital heart disease, and immunosuppression [17]. However, the most common predisposing factor in children is the presence of a congenitally abnormal pulmonary valve, and in adults is a history of intravenous drug abuse [5].

## Conclusions

This finding of a possible isolated pulmonic valve endocarditis in a patient with sickle cell disease is unique. The lack of growth of organisms of urine and blood culture and lack of predispositions to right-sided endocarditis contributed to the challenges in diagnosis. Persistent hypoxia in long-term sickle cell patients should prompt the investigation to rule out underlying pulmonary hypertension. However, this case highlights the importance of TTE and TEE in the diagnosis of suspected pulmonic valve endocarditis.
